# Answer to Commentary on “Systematic review about complementary medical hyperthermia in oncology” by Liebl et al.

**DOI:** 10.1007/s10238-022-00901-5

**Published:** 2022-10-14

**Authors:** J. Hübner, J. Dörfler, C. Liebl, L. Käsmann

**Affiliations:** 1 Klinik für Innere Medizin II; Universitätsklinikum Jena, Jena, Germany; 2 LMU Klinikum Munich, Munich, Germany

Before turning to the arguments of the authors of the letter, we would like to make the scientific reader aware of the deep conflict of interest of the authors of the letter.

The methods of hyperthermia we addressed in our systematic review, namely electrohyperthermia or whole-body hyperthermia (“alternative hyperthermia”), are methods, which are not within the lines of the internationally consented preconditions for hyperthermic treatment in oncology. At least in our country, these alternative methods are widespread and cancer patients have to pay for such treatments. Producing and selling the equipment as well as offering such treatment is a commercial business.

Secondly, the results of our systematic review are in line with the national guideline on complementary medicine (German S3 Cancer Guideline Program) which are systematically developed and based on the highest level of current evidence. The results of the search and assessment of the studies have been evaluated and discussed by the leading experts in the field in Germany and the resulting recommendation in the guideline is (translated in English):

*Data are available from 2 RCTs and 2 cohort studies on the efficacy and toxicity of electrothermia and whole-body hyperthermia in oncology patients. Electrotherapy and whole-body hyperthermia should not be performed outside of studies in these patients to reduce mortality or disease-associated or therapy-associated morbidity*. [1]

## Arguments 1–4

Considering the topic of our systematic review we made it quite clear and transparent that we only focus on what we called “complementary hyperthermia” and we defined the included treatment methods. The argument of the authors that only a more comprehensive definition of hyperthermia would be suitable for a systematic review is incorrect and an accusation of “crude methodology” only reveals that the authors’ definition on evidence-based medicine widely differs from ours. (Any systematic review on chemotherapy or any other treatment in oncology would also focus on a subset.) The subset of hyperthermic methods chosen for our review is of high interest and relevance to physicians and patients especially as they “do not meet the defined quality criteria of the European Society of Hyperthermic Oncology [Ref. 25–30 in our article]. Despite not meeting these preconditions, the methods are offered to patients and as they run under the naming of hyperthermia, even for oncologists it is hard to differentiate. Informing physicians and patients on the highly relevant difference and assessing the clinical evidence for these methods was the task in the national guideline on complementary medicine (German S3 Cancer Guideline Program [1]).

In fact, one of the authors of the commentary and author of studies included in our review writes: “Within the mEHT concept non-thermal (energy-dependent) effects are supposed to provide the dominant sensitization effects. As the temperature increase is limited and the temperature is not considered a key parameter in this concept, there is no need to measure it.” (Minnaar et al., Ref 52 in our systematic review).

Considering the terminology complementary/alternative hyperthermia: The authors of the letter use an old fashioned definition of alternative medicine as this not only comprises methods used instead of conventional treatment but also those used alongside but without evidence.

## Argument 5

The authors argue that for evidence tables, combining WBH and EH in one table is not suitable as these two methods are highly different. This once again points to some deficits in the author’s knowledge on evidence-based medicine. The criteria for assessment of risk of bias and evidence are internationally defined and only refer to the type of study. It is absolutely necessary to always apply these criteria. We agree that if we had had enough data from well-designed clinical studies to conduct a meta-analysis, using subgroups for WBH and EH would have been suitable. However, this data is missing.

## Argument 6

For all studies, we provide comprehensive data on the type of tumor and treatment. This is also the case for side effects as in the study by Fiorentine et al. [56 of the original article).

## Argument 7

The argument of selective reporting of adverse events is not valuable at all. In fact, especially in case of missing positive evidence any hint at side effects and risks is of high clinical importance for decision-making for physicians and patients.

## Argument 8

We thank the authors for their list of studies they believe we missed in our search. In fact, we considered all mentioned articles in our search but excluded them due to different reasons, we add in the table. As most probably the authors got mixed up with the reference numbers, as the Reference [2] of the Letter is an unsystematic review, we corrected the numbers (Table 1).

**Table 1:** The authors did not take the following clinical studies with EH methods into account

Malignancy	*n*	Intervention	Arms	References	Remark	Reason for exclusion
Uterus cervix	40	RT ± cHT	2	[2]	RR + ST	We completely agree with the authors of the commentary that Japanese capacitive hyperthermia is different from EHC. As a consequence, we excluded the study
Uterus cervix	110	RT ± cHT	2	[3]	RR + ST	The study was realized in an international consortium at different cities. For most cities, the exact type of hyperthermia is not stated which led to an exclusion
Uterus cervix	271	RT + ChT ± mEHT	2*	[4]	RR + ST	This study was included in our SR (Ref. 51)
Non-small-cell-lung cancer	80	RT ± cHT	2	[5]	RR + ST	Japanese capacitive hyperthermia
Non-small-cell lung-cancer	97	ChT ± mEHT ± IVC	2	[6]†	ST + QoL	In this study, patients in the intervention arm received EHT in combination with Vitamin C whereas in the control arm, they received none. As vitamin C is discussed as treatment in oncology but no final data exist, we may not derive evidence on ETH from a study only offering a combination but no arms with the singular methods. Due to this, the study was excluded
Head and neck cancer	65	RT ± cHT	2	[7]	RR	This study used a Siemens Ultraterm 607E diathermia machine, a method which is not within the scope of our review
Head and neck cancer	56	RT ± cHT	2	[8]	RR + ST	Japanese capacitive hyperthermia
Esophagus cancer	66	RT + ChT ± cHT	2	[9]	RR + ST	This study used a endoradiotherm lOOA (Olympus Co., Tokyo, Japan), a method which is not within the scope of our review
Esophagus cancer	40	ChT ± cHT	2	[10]	RR	radiofrequency system with an endotract electrode (Sugimachi et al. 1986 ~)A. thin, long electrode was placed in the oesophagus and a broad, wide counter electrode was placed on the body surface, which made the localization of the electromagnetic field feasible in the oesophagus
Bone metastases	57	RT ± cHT	2	[11]	RR	Japanese capacitive hyperthermia
Uterus cancer *(in fact the study is on cervical cancer)*	38	ChT ± mEHT	2	[12]	RR + ST	We did not include this study in our analysis as it does not meet important preconditions of conduct of a study and reporting. Despite being published in 2017, there is no ethical vote, the selection criteria of the study population are not clear as only patients who received 36 sessions of hyperthermia were selected which may lead to a high selection bias only in the intervention arm. Moreover, even if we had included this study, the result of our review would not have been different, as the study is not positive. In fact, time to relapse was 9.15 versus 8.95 months which is not clinical significant (data on statistical significance missing) and disease free survival (text in the results section) and overall survival (text for Figure 1) were not significantly longer
Urinary bladder cancer	49	RT ± cHT	2	[13]	RR + ST	Japanese capacitive hyperthermia
Peritoneal carcinomatosis	260	IPCh ± mEHT ± TCM	2	[14]†	RR	Also in this study, patients in the intervention arm received a combination of treatments (EHT and Traditional Chinese Medicine) so that the effectiveness of EHT alone cannot be assessed
Nefopam pharmacokinetics	12***	ChT ± mEHT	2	[15]	PhK	This was a pharmacokinetic study on healthy participants which is not within the scope of our review
Fentanyl pharmacokinetics	12***	ChT ± mEHT	2	[16]	PhK	This was a pharmacokinetic study on healthy participants which is not within the scope of our review
Non-small-cell-lung cancer	19	RT + cHT	1 + **	[17]	RR + ST	Japanese capacitive hyperthermia
Non-small-cell-lung cancer	35	RT + cHT	1	[18]	ST	Japanese capacitive hyperthermia
Gastric cancer	21	RT + cHT	1	[19]	RR + ST	Japanese capacitive hyperthermia
Rectal cancer	81	RT + ChT + cHT	1	[20]	RR	Japanese capacitive hyperthermia
Rectal cancer	76	RT + ChT + mEHT	1	[21]	RR + AE	In this study, the authors describe remission rates after radiochemotherapy in combination with EHT. Yet, there are no data which allow to assess the effect of EHT as no control arm exists
Rectal cancer	120	RT + ChT + mEHT	1	[22]	RR + ST	This study was included
Pediatric brain tumors	41	IT + mEHT	1	[23]	ST	In this study, children with diffuse pontine glioma received a combined treatment with Newcastle disease virus, hyperthermia, and autologous dendritic cell vaccines as part of an individualized combinatorial treatment. Accordingly, no conclusions can be drawn on the effectiveness of hyperthermia
Soft tissue sarcoma	27	RT + cHT	1	[24]	RR	Japanese capacitive hyperthermia and microwave heating apparatus
Recurrent breast cancer	26	RT + cHT	1	[25]	RR	Japanese capacitive hyperthermia and microwave heating apparatus
Brain malignancies	140	ChT ± mEHT	1	[26]†	RR + ST	This study was included
Non-small-cell lung cancer	15	ChT ± mEHT	1	[27]†	DE	This was a pharmacokinetic study on intravenous Vitamin C with patients not reporting patient relevant endpoints as defined in our review
Glioblastoma	24	ChT ± mEHT	1	[28]†	DE	This study was included

We do not understand why the authors of the commentary included the studies marked with “†—falsely interpreted” in a table of allegedly missed articles. Moreover, as there is no scientific argument for the allegedly false interpretation, this simply is an accusation and no scientific discourse which we cannot answer properly

## Argument 9

In our categorization, we followed the information in the articles. We used a highly differentiated assessment for the different study types according to internationally consented rules. (“The risk of bias in the included studies was analysed with the AMSTAR-Checklist Version 2.0 for the SR [40], the SIGN-Checklist for controlled trials Version 2.0 [41], the SIGN-Checklist for cohort studies Version 3.0 [42] and the IHE-Checklist for single-arm studies and case series [43]. (Ref refer to the reference list in our systematic review.) Again, it is not correct that evaluation of the evidence depends on the intention of those conducting the study especially not in market surveillance studies organized by those gaining profit with the method.

## Argument 10

Considering arguments in 10, we highly recommend to refer to the latest classification of level of evidence. A randomized study is not level 1b per se but only in case of high methodological quality. If this is not the case, it is considered level 2b. A minus signals, that there are some drawbacks to the study which makes it a weak candidate for the respective level. We spare the readers to discuss the accordings explanations for 11a to 11i.

## Arguments 11j

Yet, patients with advanced cancer in palliative care are an especially vulnerable group. In this case, strict adherence to evidence and avoidance of any treatment offers driven by commercial interest is ethically out of question, even if the patient may have asked for and consented to it driven by false promises. This exactly is the decisive motivation in the section on hyperthermia of the guideline. Patients must be informed on the relationship of benefits and risks.

## Arguments 11j

We thank the authors for approving our statement that there are no data on the temperature in the article. Their argument does not falsify our statement.

## Arguments 1 to 3 (we refer to the original outline number of the commentary)

As while writing our article, we were not sure whether Minnaar et al. got mixed up in their own data and reported the wrong numbers we chose a careful wording of our criticism. First of all, but only a minor point, data on HIV-treatment are missing. More important is the intransparent reporting of patient numbers. We pointed out to missing comparison of intervention and control group and missing transparency in drop out. In fact, the authors report a drop out until the inclusion in the study. The data comparison between intervention and control is made at that point for the whole remaining study group and is well-balanced.

After that, there is a substantial vanishing of patients which may lead to a high imbalance between the groups:

1. The authors start with 210 patients (Figure 1) which all received allocated intervention as they wrote. Yet, they also write “Four randomised participants did not arrive for treatment and no further data is available on these participants. They were classified as “lost to follow up”.” This is contradictory.
2. In a next step, we have 101 patients in each arm: “The intention to treat analysis showed a significant association between six month LDFS and the administration of mEHT (six month LDFS in mEHT Group: *n* = 39 [38.6%]; six month LDFS in Control Group: *n* = 20 [19.8%]; Pearson’s Chi2: *p* = 0.003).” Yet, from the text, there should be 104 in the EHT arm.
3. At six months post-treatment, 171 patients were alive (171 participants (mEHT: *n* = 88 [87.1%]; Control: *n* = 83 [82.2%]), and a PET-CT was done for 158 patients (95 EHT and 73 control). Yet, the authors calculate the percentages with 85 patients in the EHT arm (“In the mEHT Gro up (*n* = 95), there were 15[37.5%] participants with extra-pelvic visceral disease …visualised on the post-treatment 18F-FDG PET/CT scans (HIV positive: 9[23%] out of 40; HIV negative: *n* = 6[13%] out of 45).” Also in Figure 2, the authors report only 85 patients in the EHT arm, which makes a loss of an additional 10 patients from 95 which is a substantial part.

Moreover, the central outcome of the study reported on all participants is this:

“In total, 202 participants were eligible for six month LDFS analysis (mEHT: *n *= 101; Control: *n *= 101), of which 171 participants (mEHT: *n *= 88 [87.1%]; Control: *n *= 83 [82.2%]), were alive at 6 months post-treatment.”

As the authors eagerly calculate all p-values but not this one, we assume that this difference is not significant. Accordingly, this study is negative!

To provide some additional benefit for the readers of our scientific discussion, we made a quick update search of our review and screened the new data if there is any newly published article which might change the results of our SR. We found that there is none.

To summarize: there is no scientific evidence that electrohyperthermia or whole body hyperthermia has any benefit for patients beyond placebo-effects.

## References

[1] Leitlinienprogramm Onkologie (Deutsche Krebsgesellschaft, Deutsche Krebshilfe, AWMF): Komplementärmedizin in der Behandlung von onkologischen PatientInnen, Langversion 1.1, 2021, AWMF Registernummer: 032/055OL, https://www.leitlinienprogramm-onkologie.de/leitlinien/komplementaermedizin

[2] Harima Y, Nagata K, Harima K, Ostapenko VV, Tanaka Y, Sawada S (2001). A randomized clinical trial of radiation therapy versus thermoradiotherapy in stage IIIB cervical carcinoma. Int J Hyperth.

[3] Vasanthan A, Mitsumori M, Part JH (2005). Regional hyperthermia combined with radiotherapy for uterine cervical cancers: a multiinstitutional prospective randomized trial of the international atomic energy agency. Int J Rad Oncol Biol Phys.

[4] Minnaar CA, Kotzen JA, Ayeni OA (2019). The effect of modulated electro-hyperthermia on local disease control in HIV-positive and -negative cervical cancer women in South Africa: early results from a phase III randomized controlled trial. PLoS ONE.

[5] Mitsumori M, Zhi-Fan Z, Oliynychenko P (2007). Regional hyperthermia combined with radiotherapy for locally advanced non-small cell lung cancers: a multi-institutional prospective randomized trial of the International Atomic Energy Agency. Int J Clin Oncol.

[6] Ou J, Zhu X, Chen P (2020). A randomized phase II trial of best supportive care with or without hyperthermia and vitamin C for heavily pretreated, advanced, refractory non-small-cell lung cancer. J Adv Res.

[7] Datta NR, Bose AK, Kapoor HK, Gupta S (1990). Head and neck cancers: results of thermoradiotherapy versus radiotherapy. Int J Hyperth.

[8] Huilgol NG, Gupta S, Sridhar CR (2010). Hyperthermia with radiation in the treatment of locally advanced head and neck cancer: a report of randomized trial. J Cancer Res Ther.

[9] Kitamura K, Kuwano H, Watanabe M (1995). Prospective randomized stidy of hyperthermia combined with chemoradiotherapy for esophageal carcinoma. J Surg Oncol.

[10] Sugimachi K, Kuwano HL, Ide H (1994). Chemotherapy combined with or without hyperthermia for patients with oesophageal carcinoma: a prospective randomized trial. Int J Hyperth.

[11] Chi M-S, Yang K-L, Chang Y-C (2018). Comparing the effectiveness of combined external beam radiation and hyperthermia versus external beam radiation alone in treating patients with painful bony metastases: a phase 3 prospective, randomized, controlled trial. Int J Radiat Oncol Biol Phys.

[12] Lee S-Y, Lee N-R, Cho D-H (2017). Treatment outcome analysis of chemotherapy combined with modulated electro-hyperthermia compared with chemotherapy alone for recurrent cervical cancer, following irradiation. Oncol Lett.

[13] Masunaga SI, Hiraoka M, Akuta K (1994). Phase I-II trial of preoperative thermoradiotherapy in the treatment of urinary bladder cancer. Int J Hyperth.

[14] Pang CLK, Zhang X, Wang Z (2017). Local modulated electro-hyperthermia in combination with traditional Chinese medicine vs. intraperitoneal chemoinfusion for the treatment of peritoneal carcinomatosis with malignant ascites: a phase II randomized trial. Mol Clin Oncol.

[15] Lee SY, Kim M-G (2015). The effect of modulated electro-hyperthermia on the pharmacokinetic properties of nefopam in healthy volunteers: a randomised, single-dose, crossover open-label study. Int J Hyp.

[16] Lee SY, Kim M-G (2016). Effect of modulated electrohyperthermia on the pharmacokinetics of oral transmucosal fentanyl citrate in healthy volunteers. Clin Ther.

[17] Karasawa K, Muta N, Nakagawa K (1994). Thermoradiotherapy in the treatment of locally advanced nonsmall cell lung cancer. Int J Radiat Biol Phys.

[18] Ohguri T, Imada H, Yahara K (2009). Radiotherapy with 8-MHz radiofrequency-capacitive regional hypethermia for stage III non-small-cell lung cancer: the radiofrequency-output power correlates with the intraesophageal temperature and clinical outcomes. Int J Radiat Oncol Biol Phys.

[19] Nagata Y, Hiraoka M, Nishimura Y (1995). Clinical experiences in the thermoradiotherapy for advanced gastric cancer. Int J Hyperth.

[20] Shoji H, Motegi M, Takakusagi Y (2017). Chemoradiotherapy and concurrent radiofrequency thermal therapy to treat primary rectal cancer and prediction of treatment responses. Oncol Rep.

[21] You SH, Kim S (2019). Feasibility of modulated electro-hyperthermia in preoperative treatment for locally-advanced rectal cancer: early phase 2 clinical results. Neoplasma.

[22] Kim S, Lee JH, Cha J, You SH (2021). Beneficial effects of modulated electro-hyperthermia during neoadjuvant treatment for locally advanced rectal cancer. Int J Hyperth.

[23] Van Gool SW, Makalowski J, Bonner ER (2020). Addition of multimodal immunotherapy to combination treatment strategies for children with DIPG: a single institution experience. Medicines.

[24] Hiraoka M, Nishimura Y, Nagata Y (1995). Clinical results of thermoradiotherapy for soft tissue tumours. Int J Hyperth.

[25] Masunaga S, Hiraoka M, Takahashi M (1990). Clinical results of thermoradiotherapy for locally advanced and-or recurrent breast cancer—comparison of results with radiotherapy alone. Int J Hyperth.

[26] Sahinbas H, Groenemeyer DHW, Boecher E, Szasz A (2007). Retrospective clinical study of adjuvant electro-hyperthermia treatment for advanced brain-gliomas. Dtsch Z Onkol.

[27] Ou J, Zhu X, Lu Y (2017). The safety and pharmacokinetics of high dose intravenous ascorbic acid synergy with modulated electrohyperthermia in Chinese patients with stage III–IV non-small cell lung cancer. Eur J Pharm Sci.

[28] Wismeth C, Dudel C, Pascher C (2010). Transcranial electro-hyperthermia combined with alkylating chemotherapy in patients with relapsed high-grade gliomas—Phase I clinical results. J Neurooncol.

